# A Longitudinal Examination of the Social-Ecological Correlates of Exercise in Men and Women Following Cardiac Rehabilitation

**DOI:** 10.3390/jcm8020250

**Published:** 2019-02-16

**Authors:** Mahshid Moghei, Robert D. Reid, Evyanne Wooding, Gabriela Melo Ghisi, Andrew Pipe, Caroline Chessex, Stephanie A. Prince, Chris Blanchard, Paul Oh, Sherry L. Grace

**Affiliations:** 1 School of Kinesiology and Health Science, York University—Bethune 368, 4700 Keele St., Toronto, ON M3J 1P3, Canada; mmoghei@yorku.ca; 2 Division of Cardiac Prevention and Rehabilitation, University of Ottawa Heart Institute, 40 Ruskin Street, Ottawa, ON K1Y 4W7, Canada; breid@ottawaheart.ca (R.D.R.); ewooding@ottawaheart.ca (E.W.); apipe@ottawaheart.ca (A.P.); sprinceware@ottawaheart.ca (S.A.P.); 3 Faculty of Medicine, University of Ottawa, 451 Smyth Rd, Ottawa, ON K1H 8M5, Canada; Paul.Oh@uhn.ca; 4 Toronto Rehabilitation Institute, University Health Network, University of Toronto, 347 Rumsey Rd, Toronto, ON M4G 1R7, Canada; gabriela.ghisi@gmail.com (G.M.G.); caroline.chessex@uhn.ca (C.C.); 5 Department of Medicine, Dalhousie University, 5790 University Avenue, Halifax, NS B3H 1V7, Canada; chris.blanchard@dal.ca

**Keywords:** cardiac rehabilitation, cardiovascular disease, physical activity, secondary prevention, socioecological model, theory

## Abstract

Cardiac patients who engage in ≥150 min of moderate- to vigorous-intensity physical activity (MVPA)/week have lower mortality, yet MVPA declines even following cardiac rehabilitation (CR), and is lower in women. A randomized trial of nine socioecological theory-based exercise facilitation contacts over 50 weeks versus usual care (1:1 parallel arms) was undertaken (NCT01658683). The tertiary objective, as presented in this paper, was to test whether the intervention impacted socioecological elements, and in turn their association with MVPA. The 449 participants wore an accelerometer and completed questionnaires post-CR, and 26, 52 and 78 weeks later. At 52 weeks, exercise task self-efficacy was significantly greater in the intervention arm (*p* = 0.01), but no other differences were observed except more encouragement from other cardiac patients at 26 weeks (favoring controls). Among women adherent to the intervention, the group in whom the intervention was proven effective, physical activity (PA) intentions at 26 weeks were significantly greater in the intervention arm (*p* = 0.04), with no other differences. There were some differences in socioecological elements associated with MVPA by arm. There were also some differences by sex, with MVPA more often associated with exercise benefits/barriers in men, versus with working and the physical environment in women.

## 1. Introduction

Cardiovascular disease (CVD) is among the leading causes of morbidity globally [1]. Patients with CVD are at a higher risk of subsequent events, and therefore, secondary prevention is crucial. This includes physical activity, which is associated with decreased mortality and improved quality of life [2]. Patients with CVD are recommended to accumulate at least 150 min of moderate- to vigorous-intensity physical activity (MVPA) per week [3,4], by exercising for 30 min a day on most days of the week to achieve these health benefits.

However, it is known that CVD patients do not engage in sufficient MVPA [5], and that women are less active than men [6]. In the largest, most generalizable sample, it was demonstrated that only 1/3 of CVD outpatients are sufficiently active [5]. However, most data on MVPA only goes as far as 12 months after CVD hospitalization, and most studies do not assess MVPA objectively and hence are likely over-estimates [7,8,9,10,11]. It is known that cardiac rehabilitation (CR) participation is associated with greater MVPA [12]. However, MVPA also declines after graduation [13], and again the degree and course over the long-term are not well characterized.

Theoretical perspectives are key to understanding degree of MVPA, and factors that promote it; the socioecological model in particular is highly applicable [14]. It posits that patients vary in their behavior (i.e., PA) based on their individual attributes (e.g., knowledge, attitudes, and skills), as well as social (e.g., friends, family) and physical (e.g., home, neighborhood and community characteristics, weather) environments. We developed an intervention to promote PA maintenance post-CR based on this model (Ecologically-optimizing exercise maintenance in men and women post-CR; ECO-PCR) [15]. Primary outcomes of the trial are reported elsewhere [16], but in brief, the intervention did promote PA maintenance in women adherent to it. The tertiary objective of the trial was to determine whether the intervention had an effect on the socioecological model elements as planned, and if these elements were associated with MVPA over the course of the intervention and thereafter. This is tested herein, and whether this differs in men and women.

## 2. Methods

### 2.1. Design and Procedure

This was a randomized controlled, 2 parallel arm, single-blind superiority trial (clinicaltrials.gov NCT01658683). A detailed protocol is available elsewhere [15]. Tertiary outcomes are reported herein; While some comparisons are made by arm, many cross-sectional tests of associations are undertaken, as well as longitudinal observation.

Study coordinators at each site attended the second-last and final CR classes to solicit patient interest. Consenting participants were provided with a self-report survey to complete, which assessed sociodemographic characteristics, and elements of the socioecological framework. Participants were also asked to wear an ActiGraph GT3X accelerometer (ActiGraph, Pensacola, FL, USA) on their right hip using a waist belt during waking hours for nine consecutive days. Clinical data were extracted from CR charts.

Participants were stratified by recruitment site and sex, and randomized in a 1:1 ratio to either the exercise facilitator or usual care arm using a random sequence that was computer-generated by a statistical consultant in permuted blocks of 4, 8, and 10. Sequences were placed in opaque, numbered envelopes which were sealed to ensure that treatment allocation was concealed until after baseline data collection.

All participants were invited to the study centers for 3 follow-up assessments that coincided with the midpoint (26 weeks) and end-point (52 weeks) of the exercise facilitator intervention, and six months after the last contact (78 weeks). Participants were again asked to complete questionnaires to measure socioecological elements, and to wear the accelerometer. Follow-up response rates were optimized through repeated contacts. Research assistants blinded to participant allocation performed these assessments.

### 2.2. Participants and Setting

Participants were recruited from three CR programs in Ontario, Canada (one institution offered programs at two sites). Usual care consisted of provision of an updated exercise prescription and a home-based exercise program prior to program completion. Exercise maintenance strategies were reviewed. The trial was powered for the primary outcome of MVPA.

Patients were included in the study if they were currently participating in an on-site CR program of ≥8-weeks duration, graduated from CR, had a documented diagnosis of coronary artery disease (CAD), were ≥18 years old, and were able to walk unaided at 2 mph. Patients who had New York Heart Association class III or IV heart failure, were pregnant, lactating or planning to become pregnant during the study period, or were unable to read and understand English or French were excluded.

### 2.3. Intervention

The 50-week intervention was based on the socioecological model [14,17]. It consisted of one face-to-face session, five group teleconferences, three personal telephone calls, and optional visits to local exercise facilities/community exercise programs. All intervention contact was delivered by trained exercise therapists working from a standardized intervention manual. At the initial session, participants were provided an intervention workbook and a pedometer to monitor their activity against goals (and guideline recommendations). Exercise environments were reviewed. At each session, participants reviewed their activity diaries, identified barriers to exercise maintenance, and brainstormed solutions. During each telephone call, the facilitator assessed participants’ self-efficacy with respect to exercise maintenance. Barriers and solutions were discussed as appropriate, with other cardiac patients in the intervention supporting one another on group calls. Intervention fidelity was 87.40%. Intervention patients participated in a mean of 7.50 ± 2.22 of the 9 contacts (no sex differences; *p* = 0.23).

### 2.4. Measures

Sociodemographic characteristics were obtained through self-report questionnaires. Clinical characteristics of participants were obtained from CR records. The socioecological elements were assessed as outlined below.

#### 2.4.1. Individual Level

Correlates at the individual-level included many sociodemographic (i.e., sex, racial/ethnic background, work status, educational attainment, income; assessed based on [18]), and clinical characteristics (i.e., PA history, smoking status, body mass index, and comorbidities; measured based on [18]). Other individual-level correlates were assessed through psychometrically-validated, self-report scales: depressive symptoms [19], functional status [20], PA intention [21], planning [22], PA self-regulation [23], exercise task self-efficacy [22], barrier self-efficacy [21] as well as exercise benefits and barriers [24].

#### 2.4.2. Social-Environmental Level

At this level, correlates assessed were living arrangements (e.g., residing with family or not, residing with someone who requires caregiving), marital status (all prior variables assessed through investigator-generated categorical items), social support in the form of participation/involvement, providing rewards or dissuasion in the form of criticism or complaining by each of family, friends and other cardiac patients [25], subjective norms regarding PA (e.g., important others will want them engaging in MVPA [26]), and autonomy support/healthcare climate (i.e., provider encouragement of PA) [27].

#### 2.4.3. Physical-Environmental Level

The correlates at the physical-environmental level included participants’ home exercise equipment availability [28], neighborhood environment (availability of community exercise facilities [28]; as well as street connectivity, crime rate and neighborhood aesthetics) [28], season (i.e., winter months vs not) and mixed-land use (i.e., residential vs residential and commercial; the latter two assessed by investigator-generated items). The latter items were only assessed at baseline as it was assumed they would not change; except for sociodemographic characteristics, all other socioecological elements were assessed at all 4 times points (including MVPA).

#### 2.4.4. MVPA

Average weekly MVPA of participants was measured through the ActiGraph GT3X. It has been shown to be valid and reliable [29].

A 15-second sampling epoch was used and converted into 60-second epochs (counts∙min-1 (cpm)). A valid day was defined as ≥10 h of wear time, and participants were required to have a minimum of 4 valid days to be retained in the analyses. Wear time was calculated by subtracting non-wear time from 24 h. Non-wear time was defined as at least 60 min of consecutive zeros for counts, with an allowance of up to two minutes of counts between zero and 150. For participants with >7 valid days, the first day was removed (to minimize reactivity), and the subsequent 7 days used for the average.

Moderate intensity was defined using a previously-validated cut-point of ≥2690 cpm using the vector magnitude output [30]. Weekly average MVPA was calculated by multiplying the daily average minutes/day above this threshold by 7.

### 2.5. Statistical Analysis

All analyses were performed using IBM SPSS version 24 [31]. First, a descriptive examination of socioecological elements was performed, by treatment arm and sex. The association between each socioecological element and arm was assessed using *t*-tests, analysis of variance (ANOVA) or chi-square analyses, as applicable. The file was split by arm, and the association of each socioecological element and MVPA at each assessment point was tested using Spearman’s correlations, Kruskal–Wallis or Mann–Whitney tests, given MVPA was not normally-distributed. The same tests were again used to assess the association between MVPA and each of the socioecological elements at each assessment point, as applicable, in men and in women separately. A *p*-value of <0.05 was considered statistically significant.

## 3. Results

Participant accrual, randomization and retention through the trial is depicted elsewhere [16]. In summary, 2687 patients were approached, of which 1476 were deemed ineligible; 449 patients were randomized to intervention (*n* = 226) or control. Patients were considered ineligible for the following reasons: patients did not have a documented CAD diagnosis (*n* = 594; 40.24%), had attended less than 75% of the CR classes (*n* = 244; 16.53%), could not walk 2 mph to complete stress test (*n* = 242; 16.40%), did not read or understand English or French (*n* = 101; 6.84%), were planning to leave the province or region in the next 12 months (*n* = 81; 5.49%), did not graduate from CR (*n* = 66; 4.47%), were considered unable to participate in unsupervised exercise by the qualified clinical investigator (*n* = 59; 3.99%), participated in program of less than eight weeks duration (*n* = 48; 3.25%), had a New York Heart Association class III or IV (*n* = 26; 1.76%), among other reasons. Sociodemographic and clinical characteristics of participants were reported in detail by arm and retention status (retained intervention participants were significantly older and engaged in more MVPA at baseline, and retained usual care participants were significantly more educated than those lost to follow-up; no other differences observed) elsewhere [16].

Degree of MVPA at each assessment point is also depicted there, within sex and by arm [16]. In 108 (36.4%) participants (both arms), MVPA at 52 weeks was ≥MVPA at baseline (i.e., they at least maintained MVPA as per trial objective). The intervention had an impact on MVPA in women only, per protocol (i.e., those adherent to ≥2/3 personal calls and 3/5 group calls). There was also beneficial impact on secondary outcomes [16].

### 3.1. Effect of Intervention on Socioecological Elements

Table 1 shows the socioecological elements at each time point by study arm, and Table 2 shows ranges for the psychometrically-validated scales. As displayed, there appeared to be a ceiling effect for PA intention, planning, subjective norm, as well as a floor effect for exercise barriers, and PA encouragement from friends and other cardiac patients, perhaps related to the effects of CR.

Table 1 also displays socioecological elements at each time point and their association with study arm. At 26 weeks, PA participation with/encouragement by other heart patients was significantly higher in the control than the intervention arm (contrary to hypothesis). At 52 weeks, exercise task self-efficacy was significantly greater in intervention participants. At 78 weeks, there were no differences in socioecological elements by arm (but more smokers in control arm). No other differences were observed.

Given the lack of identification of impact of the intervention on theoretical constructs as intended, these associations were examined in women adherent to the intervention (i.e., attended at least 2/3 group calls and 3/5 group calls), in whom an intervention effect was observed [16]. This was a post-hoc analysis, but is consistent with the a priori focus on sex. Only the theoretical, modifiable constructs were tested. As shown in Table 2, across the 26-, 52- and 78-week assessments, the only significant difference was in PA intentions at 26 weeks (*p* = 0.04), with results supporting the intervention.

### 3.2. Association between Socioecological Elements and MVPA

Table 1 also shows the association between socioecological elements and MVPA within arm by time. When considering the more modifiable theoretical elements targeted by the intervention, in the intervention group at 26 weeks, significant associations with MVPA included greater PA intentions, planning, task and barrier self-efficacy, perceiving greater PA benefits and less barriers, availability of a bicycle, and using local recreation centres as well as indoor shopping malls (where there are walking groups before the mall opens during months of inclement weather). At 52 weeks after CR, in the intervention group, there was a positive association between each of PA intentions, planning, task and barrier self-efficacy, using a treadmill, living near community recreation centers as well as shopping malls and MVPA at that time point. At 78 weeks after CR, in the intervention group, there was a significant association between MVPA at that point and each of having greater exercise task self-efficacy, as well as having and availing themselves of a treadmill at home, having skating arenas in the neighborhood and being close to a body of water. Age, as well as work and functional status were also related in a positive direction at various time points (fairly consistent in control arm).

In the control arm, at 26 weeks, the theoretical elements associated with MVPA were similar, except PA self-regulation, subjective norm and street connectivity were associated; barrier self-efficacy, exercise benefits / barriers were not, and home and neighborhood facilities associated with MVPA differed (Table 1; the latter differences likely arbitrary). At 52 weeks post-CR, the differences in socioecological elements associated with MVPA in the control arm from the intervention arm were that there was an association with PA self-regulation, exercise benefits and barriers (negative direction), PA encouragement from friends, and again the home and neighborhood facilities associated with MVPA differed. At 78 weeks, PA self-regulation, PA intention and planning, barrier self-efficacy, exercise barriers (negative direction) and PA norms were associated with MVPA in the control arms (but not intervention), and again the home and neighborhood facilities associated with MVPA differed.

Table 3 shows socioecological elements by level, sex and assessment point. Age and sex were associated with MVPA at all time points in both sexes, in a negative and positive direction respectively. Work status (working) and annual income (positive association) were significantly associated with MVPA at all time points in women only. PA self-regulation was positively associated with MVPA in both men and women at a couple of time points. PA intentions and planning were quite consistently associated with greater MVPA at all time points except the last one in both men and women. Exercise task and barrier self-efficacy were almost universally associated with greater MVPA at all assessment points in both sexes. Exercise benefits and barriers (negative direction) were only associated with MVPA at 26 and 52 weeks post-CR in men; there were no associations for either sex at 78 weeks. 

As also shown in Table 3, factors at the social-environmental level were scarcely associated with MVPA at any time point (only encouragement from other cardiac patients was associated in men at 52 weeks). Finally, it appeared availability/use of exercise amenities in the home and neighborhood environment were associated with MVPA more often in women than men, amenities such as bikes and bike paths, weight training equipment, fitness centres, raquet clubs and bodies of water. Neighborhood aesthetics (positive association) and crime (negative) were associated with MVPA at initial assessment in women only.

## 4. Discussion

There is great need to promote MVPA to reduce the burden of CVD. Despite efforts to promote PA in CR and thereafter, impacts have been inconsistent at best, with theoretical targets remaining elusive [32]. Our intervention, based on the socioecological model, incorporating cognitive and behavioral strategies and taking into consideration patients’ sociodemographic and clinical characteristics as well as social and environmental exercise contexts, revealed very minimal impacts, disconfirming our hypotheses.

The intervention promoted PA planning, self-efficacy, self-regulation, social support, and identification of neighborhood amenities for exercise. While these constructs did differ at some assessment points by arm, the lack of intervention impact on the targeted socioecological correlates above may have been due to ceiling effects in some instances; participation in CR already impacted these constructs positively. We may also have made poor choices in the scales administered (i.e., not a good match to the intervention), or failed to consider some other important theoretical constructs. There was also some suggestion that there were more theoretical elements associated with MVPA in controls than in intervention participants, such that the intervention did impact MVPA through different mechanisms. Nevertheless, only one-third of patients were meeting guideline recommendations of ≥150 min of MVPA/week at 1-year post-CR follow-up, and the intervention was only effective in women adherent to it. A review of other interventions for exercise maintenance post-CR [33], and some trials published since [34], point us to other potential approaches or constructs to target. Ultimately, results suggest that the intervention worked in adherent women by increasing their PA intentions, however intentions in all participants were quite high across all follow-up assessments.

Results of this study are fairly consistent with other research in the field on individual-level factors related to MVPA, namely exercise task and barrier self-efficacy, PA self-regulation, planning, intentions, as well as exercise benefits/barriers (the latter particularly in men) [33]. MVPA was not highly related to the social environment, but much MVPA was also associated with the physical, built environment, particularly in women. These results, along with existing evidence [35,36], suggest we must continue to advocate to ensure environments are conducive to physical activity for the primary and secondary prevention of CVD. A variety of settings were associated with PA, from indoor mall walking to soccer pitches. Urban planners must ensure street connectivity to make it easier for patients to get around. We also must advocate with police services to promote greater neighborhood safety, so women can be active without fear. Results of this study certainly support a “health-in-all-policies” approach. Winter months are quite cold where the trial was undertaken, and it is evident that patients need suitable environments in which to exercise in both warm and cold months (e.g., mall walking, swimming pools).

Caution is warranted when interpreting these results. First, generalizability is limited to CR graduates, who likely engage in more MVPA than the average cardiac patient, and also as observed, already had high PA intentions, planned PA and reported low PA barriers. The intervention may have had a different impact in cardiac patients who do no access CR.

Second, the accelerometer cut-points used for the exercise intensities were not based on CR samples, and therefore may have misclassified some PA. There are no validated cut-points for use in the CR population. Our previous work has shown that results vary based on cut-points chosen [37].

Third and chiefly, there is likely inflated error due to multiple comparisons, however we wanted to take full consideration of the many potential impacts of the intervention and socioecological correlates of MVPA. What associations were observed may be spurious. There were few reliable associations observed, and with regard to the latter, future research is needed to replicate these to be certain they play a key role in exercise maintenance post-CR. Finally, and related, sample sizes may have been too small to the test of associations between arm and socioecological correlates in women adherent to the intervention, particularly for the later assessments points, and hence true associations may not have been identified.

## 5. Conclusions

Promoting MVPA in CVD patients, especially women, after CR remains a challenge. Our intervention showed minimal impact on socioecological elements related to PA. Some socioecological correlates of MVPA were different in men (exercise benefits/barriers) and women (work status, exercise amenities, physical environment). Overall findings highlight the importance of self-efficacy, PA intentions, planning, self-regulation and exercise benefits/barriers at the individual level, as well as in in exercise-friendly environments for MVPA in cardiac outpatients. Through also considering other intervention trials in this area, better approaches to exercise maintenance could be elucidated.

## Figures and Tables

**Table 1:** Socioecological Elements, by Level and Study Arm through 1.5 Years Following Cardiac Rehabilitation Completion and Association with Moderate and Vigorous Intensity Physical Activity.

Socioecological Elements Mean ± SD *n* (%)	Baseline (Post-CR)		26 Weeks		52 Weeks		78 Weeks	
	Control (*n* = 199)	Intervention (*n* = 204)	Control (*n* = 178)	Intervention (*n* = 172)	Control (*n* = 160)	Intervention (*n* = 153)	Control (*n* = 133)	Intervention (*n* = 120)
Individual Level								
Age	64.05 ± 9.80 ***	63.73 ± 9.96 ***	- ***	-	- ***	- **	- ***	- ***
Sex (% Male)	156 (70.00) **	158 (69.90) **	-	-	- *	-	-	-
Racial/Ethnic Background (% white/Caucasian)	174 (86.10)	179 (87.30)	-	-	-	-	-	-
Work Status (% Retired)	110 (55.00) **	105 (52.80) **	- *	- *	- *	- **	- **	- ***
Highest Education (% ≥ university)	110 (52.40)	92 (43.00)	-	-	-	-	-	-
Annual Income (≥$50,000 CAD)	103 (53.90)	102 (53.70)	-	-	-	-	-	-
Functional Status	46.88 ± 12.11 ***	46.58 ± 11.27 ***	49.87 ± 10.69 **	48.54 ± 11.43 *	49.26 ± 10.58 **	51.33 ± 8.86 *	50.00 ± 11.71 **	49.23 ± 10.09 *
Comorbidities (% yes)	181 (81.20) *	181 (80.10)	-	-	-	-	- *	-
Smoking Status (% Current)	4 (2.10)	3 (1.50)	8 (5.60)	2 (1.40)	5 (3.50)	2 (1.60)	5 (4.40)	2 (2.00)
PA Self-Regulation	3.47 ± 0.69 **	3.39 ± 0.69 *	3.37 ± 0.78 **	3.35 ± 0.76	3.36 ± 0.77 ***	3.38 ± 0.74	3.39 ± 0.76 *	3.34 ± 0.77
PA Intention	4.73 ± 0.61	4.66 ± 0.72	4.54 ± 0.66 **	4.64 ± 0.51 ***	4.51 ± 0.80 **	4.56 ± 0.70 **	4.57 ± 0.65 **	4.55 ± 0.74
PA Planning	4.44 ± 0.76 *	4.36 ± 0.82 *	4.22 ± 0.81 ***	4.35 ± 0.71 ***	4.29 ± 0.83 ***	4.37 ± 0.79 *	4.25 ± 0.75 *	4.33 ± 0.84
Task Self-Efficacy	7.58 ± 1.81 **	7.64 ± 1.87 **	8.26 ± 10.89 *	7.50 ± 1.91 ***	7.07 ± 2.12 **	7.65 ± 1.84 § ***	8.13 ± 13.29 ***	7.41 ± 2.21 *
Barrier Self-Efficacy	7.71 ± 8.15	6.99 ± 72	6.80 ± 2.43	7.37 ± 4.28 ***	6.73 ± 2.25 *	6.71 ± 2.62 **	6.86 ± 2.53 **	7.08 ± 2.33
Exercise Benefits	3.22 ± 0.32	3.17 ± 0.32 *	3.13 ± 0.43	3.12 ± 0.37 **	3.21 ± 0.35 **	3.18 ± 0.31	3.21 ± 0.35	3.15 ± 0.31
Exercise Barriers	1.73 ± 0.36 *	1.84 ± 0.4 * §§	1.86 ± 0.46	1.88 ± 0.43 **	1.80 ± 0.40 **	1.83 ± 0.38	1.90 ± 0.40 *	1.89 ± 0.37
Social-Environmental Level								
Living Status (% with Family)	151 (74.40)	153 (74.60)	-	-	-	-	-	-
Living with Someone Who Requires Caregiving	14 (6.90)	14 (6.90)	-	-	-	-	-	-
Marital Status (% Married)	149 (73.40)	147 (72.40)	-	-	-	-	-	-
Subjective Norm	4.32 ± 0.66	4.19 ± 0.76	4.21 ± 0.78 *	4.19 ± 0.70	4.25 ± 0.76	4.27 ± 0.67	4.24 ± 0.69*	4.20 ± 1.27
Healthcare Climate	5.33 ± 1.55	5.25 ± 1.63	4.74 ± 1.69	5.05 ± 1.85	4.84 ± 1.74	4.93 ± 1.85	4.39 ± 1.80	4.67 ± 1.90
Social Support for PA								
Encouragement from Family	2.44 ± 1.44	2.59 ± 1.52	2.46 ± 1.47	2.21 ± 1.43	2.40 ± 1.51	2.29 ± 1.40	2.20 ± 1.40	2.32 ± 1.40
Encouragement from Friends	1.55 ± 1.02 *	1.82 ± 1.28	1.91 ± 1.31	1.85 ± 1.23	2.15 ± 1.39 *	1.95 ± 1.24	1.89 ± 1.24	1.98 ± 1.25
Encouragement from Other Cardiac Patients	1.43 ± 0.97	1.43 ± 0.92	1.51 ± 1.16 §§	1.14 ± 0.55	1.42 ± 1.08	1.20 ± 0.61	1.35 ± 0.93	1.16 ± 0.57
Family Rewarding PA	1.47 ± 0.98	1.42 ± 0.90	1.42 ± 0.83	1.35 ± 0.80	1.43 ± 0.93	1.41 ± 0.83	1.47 ± 0.98	1.40 ± 0.83
Friend Rewarding PA	1.05 ± 0.31	1.16 ± 0.58	1.15 ± 0.48	1.10 ± 0.40	1.14 ± 0.61	1.13 ± 0.47	1.17 ± 0.62	1.19 ± 0.60
Other Cardiac Patients Rewarding PA	1.02 ± 0.12	1.04 ± 0.22	1.05 ± 0.28	1.03 ± 0.20	1.09 ± 0.50	1.03 ± 0.23	1.07 ± 0.42	1.01 ± 0.11
Family Dissuading PA	1.05 ± 0.37	1.04 ± 0.24	1.10 ± 0.42	1.04 ± 0.24	1.05 ± 0.35	1.08 ± 0.41	1.06 ± 0.41	1.02 ± 0.16
Friend Dissuading PA	1.01 ± 0.11	1.04 ± 0.30	1.08 ± 0.44	1.03 ± 0.24	1.04 ± 0.34	1.04 ± 0.19	1.03 ± 0.17	1.01 ± 0.14
Other Cardiac Patient Dissuading PA	1.02 ± 0.12	1.01 ± 0.08	1.01 ± 0.09	1.00 ± 0.00	1.00 ± 0.00	1.01 ± 0.10	1.14 ± 1.07	1.01 ± 0.11
Physical-Environmental Level								
Home Resources for PA ‡			-	-	-	-	-	-
Treadmill	73 (36.70)	62 (30.70)	-	-	-	- ¶	-	- † ¶
Stationary Bike	59 (29.90)	81 (40.50) †	-	-	-	-	-	-
Outdoor Bicycle	101 (51.50)	118 (59.60)	-	- †	-	-	-	- †
Ski	69 (35.20)	62 (31.20)	-	-	-	-	-	-
Skate	89 (45.60)	87 (43.70) ¶	-	-	-	-	-	-
Weight Train. Equip	110 (55.30)	107 (53.80) ¶	-	-	- ††	-	- ¶	-
Running Shoes	199 (100.00)	196 (97.50)	-	-	-	-	-	-
Swimming Pool	38 (19.10)	49 (24.90)	-	-	-	-	-	-
Toning Devices	126 (64.00)	114 (57.00)	-	-	-	-	-	-
Aerobic PA Video	56 (28.40)	38 (19.20)	-	-	-	-	-	-
Dog	53 (26.60)	39 (19.80)	-	-	-	-	-	-
Neighborhood Characteristics								
Aesthetics	3.43 ± 0.58	3.49 ± 0.76 **	-	-	-	-	-	-
Crime Rate	1.23 ± 0.41	1.20 ± 0.36	-	-	-	-	-	-
Street Connectivity	3.01 ± 0.80	3.06 ± 0.81	- *	-	-	-	-	-
Places to do Physical Activity in Community ‡			-	-	-	-	-	-
Fitness Clubs	170 (85.00)	165 (81.70)	-	-	-	-	-	-
Schools with Rec. Prog	108 (55.40) †	99 (53.20)	-	- ¶	-	-	- †	-
Community Rec. Cen.	150 (76.50)	151 (77.80) †	-	- ¶¶	-	- †	-	-
Skating/Hockey Arenas	143 (73.30)	144 (73.50)	-	-	-	-	-	- †
Jogging/Walking Paths	179 (89.90)	179 (89.10)	- ¶	-	- ¶	-	- ¶	-
Bicycle Lanes/Paths	163 (82.30)	164 (82.40) †	-	-	-	-	-	-
Swimming Pools	155 (77.50)	155 (77.10)	-	-	- ¶	-	-	-
Racquet Clubs	136 (70.50) ¶	137 (69.50)	-	-	-	-	-	-
Indoor Shopping Mall	134 (67.00)	128 (63.10)	-	- ¶	-	- †	-	-
Golf Course	105 (52.80)	92 (46.00)	-	-	-	-	-	-
Beaches/Lakes/River/Creek	110 (55.00) ¶¶	101 (49.80) †	-	-	-	-	- ¶¶	- †
Public Parks	194 (96.50)	190 (93.10)	-	-	-	-	-	-
Soccer/Football Field	173 (89.60) ††	167 (82.70)	- † ¶¶	-	- ††	-	- ¶ †	-

CR, cardiac rehabilitation; MVPA, moderate- to vigorous-intensity physical activity; PA, physical activity; SD, standard deviation; CAD, Canadian dollars; - not assessed at this time point. ‡ frequency reporting availability; § *p* < 0.05, §§ *p* < 0.01 for differences by arm; * *p* < 0.05, ** *p* < 0.01, *** *p* < 0.001 assessing association between amount of MVPA at same assessment point and socioecological element score at same assessment point, within arm. For those elements not assessed after the initial assessment, the association of the initial score with MVPA at each assessment point is shown where significant. Association between amount of MVPA at same assessment point and availability † *p* < 0.05, †† *p* < 0.01; Association between amount of MVPA at same assessment point and use of element ¶ *p* < 0.05, ¶¶ *p* < 0.01.

**Table 2:** Socioecological Elements, by Level and Study Arm through 1.5 Years Following Cardiac Rehabilitation Completion in Women Adherent to the Intervention.

Socioecological Elements Mean ± SD *n* (%)	Score Range	Baseline (Post-CR)		26 Weeks		52 Weeks		78 Weeks	
		Control (*n* = 61)	Intervention (*n* = 63)	Control (*n* = 54)	Intervention (*n* = 49)	Control (*n* = 48)	Intervention (*n* = 46)	Control (*n* = 44)	Intervention (*n* = 39)
Individual Level									
PA Self-Regulation	1–5	3.63 ± 0.60	3.40 ± 0.66	3.51 ± 0.84	3.39 ± 0.73	3.50 ± 0.81	3.33 ± 0.84	3.47 ± 0.82	3.38 ± 0.77
PA Intention	1–5	4.73 ± 0.63	4.62 ± 0.78	4.43 ± 0.72	4.68 ± 0.45 §	4.64 ± 0.59	4.58 ± 0.53	4.56 ± 0.77	4.57 ± 0.74
PA Planning	1–5	4.47 ± 0.80	4.33 ± 0.83	4.18 ± 0.79	4.43 ± 0.66	4.45 ± 0.76	4.36 ± 0.61	4.26 ± 0.80	4.31 ± 0.88
Task Self-Efficacy	1–10	7.67 ± 1.54	7.54 ± 1.93	7.46 ± 1.72	7.85 ± 2.06	7.38 ± 2.05	7.55 ± 2.06	7.16 ± 2.12	7.66 ± 1.85
Barrier Self-Efficacy	0–10	7.21 ± 1.94	6.85 ± 1.96	6.79 ± 2.04	7.54 ± 5.09	6.77 ± 2.33	6.74 ± 2.11	6.89 ± 2.60	7.08 ± 1.84
Exercise Benefits	1–4	3.19 ± 0.33	3.11 ± 0.33	3.21 ± 0.32	3.10 ± 0.37	3.19 ± 0.31	3.14 ± 0.29	3.19 ± 0.34	3.14 ± 0.27
Exercise Barriers	1–4	1.79 ± 0.39	1.91 ± 0.38	1.81 ± 0.39	1.91 ± 0.40	1.84 ± 0.35	1.88 ± 0.32	2.03 ± 0.42	1.98 ± 0.39
Social-Environmental Level									
Subjective Norm	1–5	4.31 ± 0.76	4.16 ± 0.76	4.24 ± 0.74	4.35 ± 0.53	4.40 ± 0.49	4.28 ± 0.48	4.24 ± 0.85	4.16 ± 0.88
Healthcare Climate	1–7	5.26 ± 1.69	5.01 ± 1.79	4.71 ± 1.72	5.05 ± 1.89	4.78 ± 2.00	4.54 ± 2.06	4.66 ± 1.99	4.02 ± 2.20
Social Support for PA									
Encouragement from Family	1–5	2.21 ± 1.08	2.21 ± 1.01	2.23 ± 1.07	2.21 ± 1.03	2.25 ± 1.19	2.20 ± 1.19	2.27 ± 1.15	2.15 ± 1.00
Encouragement from Friends	1–5	1.75 ± 0.87	1.85 ± 1.02	1.66 ± 0.84	1.66 ± 0.85	1.77 ± 0.85	1.90 ± 1.04	1.80 ± 1.00	1.87 ± 1.02
Encouragement from Other Cardiac Patients	1–5	1.25 ± 0.50	1.29 ± 0.59	1.40 ± 0.85	1.12 ± 0.36	1.23 ± 0.52	1.18 ± 0.41	1.46 ± 0.41	1.09 ± 0.27
Family Dissuading PA	1–5	2.06 ± 0.31	2.08 ± 0.56	2.02 ± 0.34	2.00 ± 0.31	1.08 ± 0.30	1.06 ± 0.25	1.04 ± 0.18	1.07 ± 0.25
Friend Dissuading PA	1–5	2.05 ± 0.40	2.07 ± 0.53	2.04 ± 0.49	1.97 ± 0.34	1.03 ± 0.16	1.02 ± 0.10	1.09 ± 0.32	1.02 ± 0.16
Other Cardiac Patient Dissuading PA	1–5	2.00 ± 0.00	1.98 ± 0.13	1.97 ± 0.41	2.03 ± 0.33	1.00 ± 0.00	1.00 ± 0.00	1.04 ± 0.20	1.00 ± 0.00

CR, cardiac rehabilitation SD, standard deviation; § *p* < 0.05 for differences by arm.

**Table 3:** Socioecological Elements, by Level, and Association with Moderate and Vigorous Intensity Physical Activity Through 1.5 Years Following Cardiac Rehabilitation Completion by Sex.

Socioecological Elements Mean ± SD or *n* (%)	Baseline (Post-CR)		26 Weeks		52 Weeks		78 Weeks	
	Men (*n* = 279)	Women (*n* = 124)	Men (*n* = 247)	Women (*n* = 103)	Men (*n* = 219)	Women (*n* = 94)	Men (*n* = 170)	Women (*n* = 83)
Individual Level								
Age	63.10 ± 9.56 ***	65.72 ± 10.37 ***	- **	- ***	- ***	- ***	- ***	- ***
Racial/Ethnic Background (% White/Caucasian)	242 (85.20)	111 (90.20)	-	-	-	-	- **	-
Work Status (% Retired)	133 (48.00)	82 (67.20) ***	-	- **	-	- ***	-	-**
Highest Education (% ≥ University)	143 (50.70)	59 (48.40)	-	-	-	-	-	-
Annual Income ≥$50,000 CAD	161 (60.10)	44 (38.90)	-	- *	-	- *	-	-*
Functional Status §	48.37 ± 10.59 ***	41.99 ± 13.35 **	50.87 ± 9.52 **	45.01 ± 13.40 *	51.04 ± 9.17 **	48.13 ± 11.19 *	51.26 ± 9.43 **	45.57 ± 13.42 **
Comorbidities (% Yes)	243 (77.40)	119 (88.10)	-	-	-	-	- **	-
Smoking Status (% Current)	5 (1.80)	2 (1.80)	70 (36.50)	40 (45.50)	62 (33.90)	29 (36.30)	9 (6.00)	2 (3.10)
PA Self-Regulation	3.40 ± 0.71 **	3.51 ± 0.64	3.31 ± 0.76 **	3.45 ± 0.79	3.34 ± 0.72 ***	3.42 ± 0.82 **	3.34 ± 0.75	3.43 ± 0.79 **
PA Intention	4.70 ± 0.65	4.67 ± 0.71 **	4.61 ± 0.57 ***	4.54 ± 0.62 **	4.50 ± 0.82 **	4.62 ± 0.56 ***	4.56 ± 0.67	4.58 ± 0.75 *
PA Planning	4.40 ± 0.78 *	4.40 ± 0.82 *	4.28 ± 0.72 ***	4.29 ± 0.75 **	4.30 ± 0.86 **	4.40 ± 0.69 **	4.29 ± 0.78	4.28 ± 0.83
Task Self-Efficacy	7.62 ± 1.88 **	7.60 ± 1.74 **	7.40 ± 1.81 ***	8.98 ± 13.95 **	7.30 ± 1.99 ***	7.46 ± 2.04 ***	7.98 ± 11.78 **	7.40 ± 2.00 **
Barrier Self-Efficacy	7.09 ± 2.38	7.97 ± 10.45 *	7.05 ± 3.33 **	7.16 ± 3.83 *	6.70 ± 2.53 *	6.76 ± 2.21 *	6.96 ± 2.51 *	6.98 ± 2.27 *
Exercise Benefits	3.21 ± 0.32	3.15 ± 0.33	3.11 ± 0.42 **	3.16 ± 0.35	3.21 ± 0.34 **	3.17 ± 0.30	3.18 ± 0.34	3.17 ± 0.32
Exercise Barriers	1.76 ± 0.38	1.85 ± 0.39 *	1.87 ± 0.46 **	1.86 ± 0.39	1.79 ± 0.42 *	1.86 ± 0.34	1.84 ± 0.37	2.00 ± 0.40
Social-Environmental Level								
Living Status (% with Family)	232 (81.70)	72 (58.10)	-	-	-	-	-	-
Living with Someone Who Requires Caregiving (% Yes)	19 (6.70)	9 (7.30)	-	-	-	-	- *	-
Marital Status (% Married)	229 (81.20)	67 (54.00)	-	-	-	-	-	-
Subjective Norm	4.26 ± 0.69	4.23 ± 0.76	4.16 ± 0.77	4.29 ± 0.65	4.23 ± 0.80	4.34 ± 0.49	4.23 ± 1.1	4.21 ± 0.86
Healthcare Climate	5.36 ± 1.53	5.13 ± 1.74	4.91 ± 1.77	4.87 ± 1.81	4.98 ± 1.68	4.67 ± 2.02	4.59 ± 1.73	4.37 ± 2.11
Social Support for PA								
Encouragement from Family	23.77 ± 10.62	20.04 ± 9.58	2.39 ± 1.45	2.18 ± 1.46	2.39 ± 1.42	2.24 ± 1.53	2.28 ± 1.41	2.20 ± 1.37
Encouragement from Friends	16.35 ± 9.66	15.75 ± 7.55	1.89 ± 1.29	1.87 ± 1.23	2.07 ± 1.35	2.01 ± 1.25	1.85 ± 1.21	2.10 ± 1.29
Encouragement from Other Cardiac Patients	12.25 ± 12.47	12.80 ± 14.95	1.75 ± 5.93	1.38 ± 1.07	1.32 ± 0.91 *	1.29 ± 0.80	1.30 ± 0.87	1.16 ± 0.48
Family Rewarding PA	1.45 ± 0.95	1.32 ± 0.83	1.41 ± 0.86	1.30 ± 0.67	1.48 ± 0.92	1.30 ± 0.79	1.53 ± 1.00	1.20 ± 0.59
Friend Rewarding PA	1.12 ± 0.59	1.07 ± 0.39	1.13 ± 0.49	1.10 ± 0.33	1.15 ± 0.59	1.11 ± 0.44	1.22 ± 0.64	1.10 ± 0.51
Other Cardiac Patients Rewarding PA	1.01 ± 0.16	1.01 ± 0.13	1.04 ± 0.25	1.03 ± 0.24	1.07 ± 0.42	1.04 ± 0.28	1.04 ± 0.32	1.06 ± 0.33
Family Dissuading PA	2.20 ± 0.95	2.07 ± 0.45	1.09 ± 0.40	1.00 ± 0.00	1.06 ± 0.42	1.06 ± 0.27	1.05 ± 0.36	1.02 ± 0.16
Friend Dissuading PA	2.03 ± 0.41	2.06 ± 0.47	1.07 ± 0.40	1.02 ± 0.20	1.05 ± 0.33	1.01 ± 0.10	1.01 ± 0.11	1.05 ± 0.22
Other Cardiac Patient Dissuading PA	2.01 ± 0.58	1.99 ± 0.96	1.00 ± 0.00	1.01 ± 0.12	1.01 ± 0.08	1.00 ± 0.00	1.11 ± 0.93	1.02 ± 0.15
Physical-Environmental Level								
Home resources for PA ‡								
Treadmill	97 (34.90)	38 (30.90) † ¶	-	-	-	-	-	-
Stationary Bike	106 (38.70)	34 (27.60)	-	- †	-	-	-	-
Outdoor Bicycle	172 (62.50)	47 (39.50) †	-	-	-	- †	-	- †
Ski	101 (36.70)	30 (25.00)	-	-	-	-	-	-
Skate	141 (51.50)	35 (29.20)	-	-	-	-	-	-
Weight Train. Equip	146 (52.70)	71 (58.70)	-	- †† ¶	-	-	-	- ¶
Running Shoes	273 (98.20)	122 (100.00)	-	-	-	-	-	-
Swimming Pool	63 (22.90)	24 (19.80)	-	-	-	-	-	-
Toning Devices	155 (56.40)	85 (69.70) †	-	-	-	-	-	-
Aerobic Ex. Video	57 (20.90)	37 (30.30)	-	-	-	-	-	-
Dog	64 (23.20)	28 (23.30)	-	-	-	-	-	-
Neighborhood Characteristics								
Aesthetics	3.42 ± 0.59	3.56 ± 0.83 **	-	-	-	-	-	-
Crime Rate	1.17 ± 0.34	1.31 ± 0.46 *	-	-	-	-	-	-
Street Connectivity	3.01 ± 0.79	3.09 ± 0.83	-	-	-	-	-	-
Places to Do Physical Activity in Community ‡								
Fitness Clubs	232 (82.90)	103 (84.40)	-	- ¶	-	-	-	-
Schools with Rec. Prog	148 (55.80)	59 (50.90)	- ¶	-	-	-	-	-
Community Rec. Cen.	203 (74.90)	98 (82.40)	-	-	-	-	-	-
Skating/Hockey Arenas	204 (75.60)	83 (68.60)	-	-	-	-	-	-
Jogging/Walking Paths	255 (91.40)	103 (85.10)	-	-	-	-	-	-
Bicycle Lanes/Paths	233 (84.40)	94 (77.70) ¶	-	-	-	- †	-	-
Swimming Pools	213 (76.30)	97 (79.50)	-	-	-	-	-	-
Racquet Clubs	195 (71.40)	78 (66.70) †		-	-	- ††	-	- †
Indoor Shopping Mall	182 (64.80)	80 (65.60)	-	- †	-	-	-	-
Golf Course	145 (52.00)	52 (43.30)	-	-	- †	-	-	-
Beaches/Lakes/River/Creek	153 (54.40)	58 (47.50) ¶¶	-	- ¶	-	- ¶	-	- ¶¶
Public Parks	271 (96.10)	113 (91.90)	-	-	-	-	-	-
Soccer/Football Field	242 (86.40)	98 (81.00) †	-	-	-	-	-	-

CR, cardiac rehabilitation; MVPA, moderate- to vigorous-intensity physical activity; PA, physical activity; SD, standard deviation; -not assessed at this time point. § The Duke Activity Status Index Score [20]; ‡ frequency reporting availability, * *p* < 0.05, ** *p* < 0.01, *** *p* < 0.001 assessing association between amount of MVPA at same assessment point and socioecological element score at same assessment point, within sex. For those elements not assessed after the initial assessment, the association of the initial score with MVPA at each assessment point is shown where significant. Association between amount of MVPA at same assessment point and availability † *p* < 0.05, †† *p* < 0.01; Association between amount of MVPA at same assessment point and use of element ¶ *p* < 0.05, ¶¶ *p* < 0.01.
